# Anti-IL-6R Ab tocilizumab to treat paraneoplastic inflammatory syndrome of solid cancers

**DOI:** 10.1016/j.esmoop.2024.104088

**Published:** 2025-01-03

**Authors:** J.-Y. Blay, M. Brahmi, A. Dufresne, A. Swalduz, V. Avrillon, S. Assaad, C. Decroisette, B. Mastroianni, M. Dupont, F. Bourbotte-Salmon, I. Ray-Coquard, P. Meeus, A. Dutour, M. Castets, M. Perol, P. Heudel

**Affiliations:** 1 Department of Medicine and DRCI, Centre Léon Bérard & Université Claude Bernard Lyon I, Lyon, France; 2 Centre de Recherche en Cancérologie de Lyon, Equipe 4, Lyon, France

**Keywords:** IL-6, paraneoplastic inflammatory syndrome, tocilizumab, sarcoma, lung cancer

## Abstract

**Background:**

Paraneoplastic inflammatory syndrome (PIS) with fever and biological inflammation is a rare but severe condition often caused by the systemic production of interleukin 6 (IL-6) by cancer cells. We report on the efficacy of tocilizumab, an anti-IL-6 receptor antibody, in 35 patients with severe PIS.

**Patients and methods:**

All 35 patients with solid cancers (sarcomas, lung carcinoma, and breast carcinoma) diagnosed with a PIS from 2019 to 2024 treated with tocilizumab were analyzed in this single-center study (health authorities’ approval R201-004-478). Patients’ characteristics and clinical and biological effects of tocilizumab administration are presented.

**Results:**

Thirty-five (97%) patients had paraneoplastic fever. The median performance status (PS) was 2 (range 1-4). Forty percent of patients had lost 10% of body weight. All had increased serum C-reactive protein (CRP) levels (median 212 mg/l), and 74% and 48% had increased polymorphonuclear leukocyte (PMN) and platelet counts, respectively. Ninety-four percent had inflammatory anemia. Tocilizumab was given once in 23 (66%) patients and more than once in 12 patients. All patients experienced resolution of paraneoplastic fever, and 11 (31%) had improved PS. CRP, PMN, and platelet count decreases were observed in 100%, 85%, and 94% of patients, respectively. Seventy-seven percent of patients had increased hemoglobin levels. CRP and inflammatory symptoms often relapsed 4-6 weeks after tocilizumab in patients receiving only one injection.

**Conclusions:**

Tocilizumab is an efficient treatment for severe PIS providing significant improvement in clinical symptoms and biological abnormalities.

## Highlights

• PIS with fever and inflammation is a severe condition associated with cancer.
• PIS is often caused by the systemic production of IL-6 by cancer cells.
• Tocilizumab, an anti-IL-6 receptor antibody, was administered in 35 patients with PIS.
• All patients had paraneoplastic fever, increased serum CRP levels, and a biological inflammatory syndrome.
• Tocilizumab provided a consistent and significant improvement in clinical and biological inflammation symptoms.

## Introduction

Paraneoplastic inflammatory syndrome (PIS) is a rare but severe condition associated with solid cancers, with negative prognostic implications.^1^, ^2^, ^3^, ^4^, ^5^, ^6^, ^7^, ^8^, ^9^, ^10^, ^11^, ^12^, ^13^, ^14^, ^15^ PIS is diagnosed after the exclusion of infection and other causes of inflammation. It is mediated by the release of cytokines, hormones, or other immune-modulating factors by cancer cells.^1^ Clinical symptoms include fever, fatigue, night sweats, decreased performance status (PS), weight loss, and cachexia. Biological symptoms include C-reactive protein (CRP) increase, neutrophilia, with occasional leukemoid reactions, thrombocytosis, and inflammatory anemia.^1^, ^2^, ^3^, ^4^, ^5^, ^6^, ^7^, ^8^, ^9^, ^10^, ^11^, ^12^, ^13^, ^14^, ^15^

The overproduction of interleukin 6 (IL-6) by solid cancer cells has been reported to be involved in the pathogenesis of these systemic clinical and biological symptoms.^4^^,^^6^^,^^15^, ^16^, ^17^, ^18^, ^19^ IL-6 is a multifunctional cytokine that plays a pivotal role in immune responses, inflammation, and hematopoiesis.^18^, ^19^, ^20^, ^21^ Under normal physiological conditions, IL-6 is involved in the acute-phase response to infections and tissue injuries, facilitating the activation and differentiation of B and T cells, as well as the production of acute-phase proteins by the liver. However, in the context of paraneoplastic syndromes, the deregulated IL-6 production by cancer cells yields increased blood concentrations and systemic symptoms of inflammation.^1^^,^^4^^,^^6^ The persistent elevation of IL-6 in the bloodstream may cause fever, fatigue, weight loss, and cachexia, significantly impacting the patient’s quality of life.

Cancers causing IL-6-mediated PIS are often hematological malignancies, lymphoma, or multiple myeloma.^22^, ^23^, ^24^ Many different solid cancers are also associated with PIS: renal cell carcinomas,^1^^,^^4^^,^^6^ lung cancers,^1^^,^^2^ sarcomas,^1^^,^^7^^,^^10^ and others.^1^^,^^8^, ^9^, ^10^ In these patients, IL-6 and CRP levels are highly correlated and their increase is correlated to a worse progression-free survival and/or in a variety of solid cancers.^1^, ^2^, ^3^^,^^16^^,^^17^^,^^25^

The management of IL-6-mediated PIS requires an efficient anti-neoplastic treatment. The primary strategy is the management of the underlying malignancy, which, if successfully treated, may lead to the resolution of the inflammatory syndrome. Anti-inflammatory agents and corticosteroids may also be used to control inflammation, although their efficacy is inconsistent and often short lasting, while long-term use is associated with significant side-effects.^26^ In early studies, monoclonal anti-IL-6 antibodies (Abs) were reported to improve clinical and biological symptoms of PIS in patients with solid and hematological tumors.^6^^,^^22^, ^23^, ^24^ In case reports, tocilizumab, a monoclonal antibody against the IL-6 receptor (IL-6R), has also been shown to be effective in reducing the inflammatory symptoms and improving the quality of life in affected patients.^27^^,^^28^

We report here a series of 35 patients describing the efficacy of tocilizumab in PIS associated with solid cancers.

## Patients and methods

### Patients

Patients treated with tocilizumab at Centre Léon Bérard (CLB) since 2019 were included in a retrospective study conducted according to the methodology MR004 of the French National Health authorities. The study was approved in August 2024 under the number R201-004-478. Inclusion criteria were a diagnosis of PIS excluding other causes of systemic inflammation, and the administration of tocilizumab to treat these symptoms. Only patients who agreed to the reuse of their data could be included.

### Criteria used for the selection of patients

There is no universally accepted definition of PIS but several characteristics have to be met. The following criteria were used in this study to qualify the PIS of these patients: (i) absence of documented infection, (ii) duration ≥3 weeks, (iii) absence of associated inflammatory or immune condition, (iv) fever >38°C and/or chronic night sweats, and/or weight loss, (v) increased CRP level (without threshold levels).

### Clinical and biological data

The data were collected using the CONSORE tool from the electronic patient records of CLB.^29^, ^30^, ^31^ Eighty-five patients were treated with tocilizumab at CLB between 2019 and 2024. Nine were treated for a coronavirus diease-19 pneumonia, 41 received tocilizumab for cytokine release syndrome (CRS) after chimeric antigen receptor T-cell (CAR-T-cell) therapy, and 35 were treated with tocilizumab for a documented PIS, with paraneoplastic fever and increased CRP levels (Table 1). Symptoms of PIS were considered as severe by the physician, because of an impact on PS (>2), weight loss >10%, and/or persistent fever. Patient characteristics, treatments, and biological results were extracted and checked manually by two researchers (J-YB, PH). Clinical (age, sex, histological type, PS, weight, treatment, etc.) and biological [CRP, polymorphonuclear leukocyte (PMN), platelet, and hemoglobin (Hb)] characteristics of all patients collected at baseline, before, during, and after tocilizumab were extracted, verified, and validated. Baseline plasma IL-6 levels were documented in 13 patients. Survival and follow-up were collected.

**Table 1:** Description of the patients

Characteristics	*n* (%)
Sex	
Female	12 (34)
Male	23 (66)
Age, median (range), years	59 (15-88)
Cancer type	
Sarcoma^a^	22 (63)
DDLPS	10 (28)
Lung cancer^b^	12 (22)
Adenocarcinoma	6 (11)
Breast cancer	1 (3)
Clinical stage before tocilizumab	
2^c^	6 (17)
4	29 (83)
Paraneoplastic fever	
Yes	34 (97)
No	1 (3)
PS	
1	4 (11)
2	14 (40)
3	12 (34)
4	4 (11)
Previous treatments	25 (71)
Context of tocilizumab administration	
Before local treatment	6 (17)
Single-agent palliative tocilizumab	6 (17)
Combination with CT	17 (44)
Combination with IO agents	6 (17)
IL-6 levels (pg/ml)	141 (56-680)
CRP levels (mg/l)	212 (32-470)
PMN count >7000 G/L	26 (74)
PLT count >450 G/L	17 (49)
Anemia	33 (94)
Number of tocilizumab courses	
1	23 (66)
2	3 (9)
3	5 (14)
4	3 (9)
25	1 (3)

AFH, angiomatoid fibrous histiocytoma; CT, chemotherapy; DDLPS, dedifferentiated liposarcoma; IL-6, interleukin 6; IO, immuno-oncologic; LMS, leiomyosarcoma; MPNST, malignant peripheral nerve sheath tumors; PLT, platelet, PMN, polymorphonuclear leukocyte; PS, performance status; RMS, rhabdomyosarcoma; UPS, undifferentiated pleomorphic sarcoma; WDLPS, well-differentiated liposarcoma.

a *n* = 4 epidermoid carcinoma, *n* = 1 undifferentiated carcinoma, *n* = 1 sarcomatoid carcinoma.

b UPS: *n* = 3, WDLPS: *n* = 3, SFT, angiosarcoma, pleomorphic RMS, LMS, AFH, MPNST *n* = 1 each.

c All stage 2 were sarcomas: *n* = 4 DDLPS, *n* = 1 UPS and angiosarcoma each.

### Tocilizumab administration

Tocilizumab was administered intravenously (i.v.) at a dose of 8 mg/kg in a short 60-min i.v. infusion once in 23 patients and every 15 days three times and then monthly in 8 patients. Tocilizumab was given every 4 weeks in four patients.

### Ethical approval and health authorities’ approval

The study was approved by the local study committee and by the national authorities according to the MR004 procedure (https://www.cnil.fr/fr/declaration/methodologie-de-reference-04-recherches-nimpliquant-pas-la-personne-humaine-etudes-et-evaluations-dans-le-domaine-de-la-sante) in August 2024 (authorization number R201-004-478).

### Statistical analysis

The distribution of clinical and biological characteristics was analyzed using the chi-square test, Fisher’s exact test, and Mann–Whitney *U* test. Survival was plotted from the date of tocilizumab injection to the date of death or to the date of last news if alive at the time of the analysis (August 2024). Survival was plotted according to the Kaplan–Meier method, and groups were compared using the log-rank test. All statistical analyses were carried out using SPSS 23.0 software (IBM, Paris, France).

## Results

### Patients

From March 2019 to July 2024, 35 patients presenting with a PIS were treated with tocilizumab at CLB (Table 1). All patients had been pretreated with paracetamol and nonsteroidal anti-inflammatory drugs with transient and incomplete efficacy on clinical inflammatory symptoms. Symptoms of PIS were considered as severe (PS >2, persistent fever, weight loss >10%) by the physician and were reported at least 3 weeks before tocilizumab in all patients. Sarcoma and lung cancers were the most common cancers in this series. All but one (*n* = 34, 97%) patient had paraneoplastic fever and night sweats. One had only night sweats. Twenty-five (60%) lost weight in the past 6 months: 15 (40%) lost >5% and 9 (26%) lost 10% of body weight.

Six (17%) patients, all sarcomas, had localized untreated disease at the date of tocilizumab injection. Six (17%) patients received tocilizumab after progression under standard treatments in the advanced phase without specific cancer therapy as palliative care. Twenty-three (68%) patients received tocilizumab in combination with systemic cancer therapies [chemotherapy *n* = 17, programmed cell death protein 1 (PD-1)/programmed death-ligand 1 (PD-L1) Ab *n* = 6]; these 23 patients received tocilizumab before (*n* = 21, median 17 days, range 1-118 days), or concomitantly (*n* = 2) to systemic cancer therapy. Ten of the 23 patients had received previous systemic therapies.

**Figure 1:**
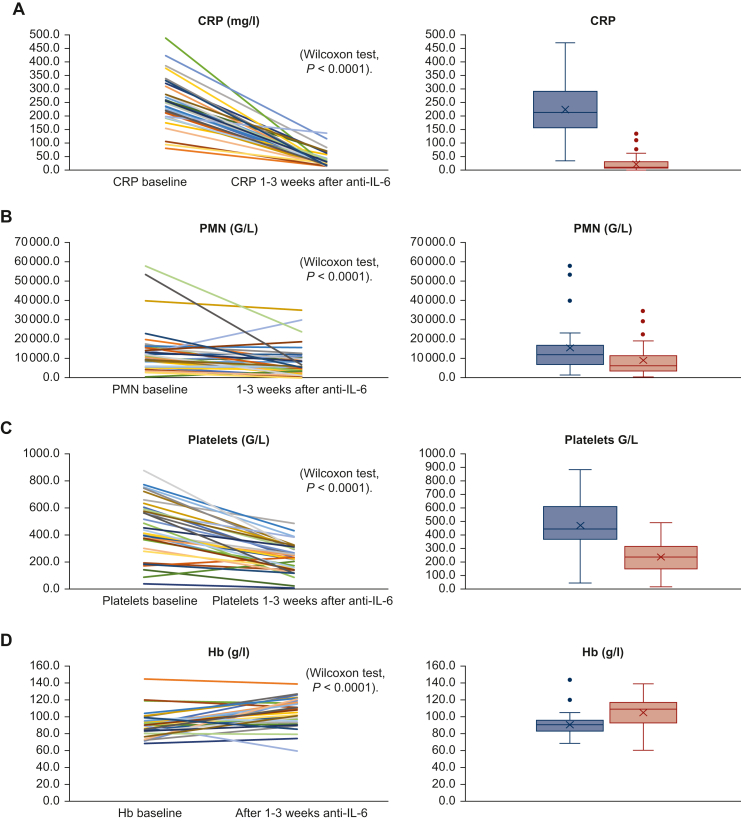
Biological inflammatory syndrome 1-3 weeks after the first injection of tocilizumab. (A) CRP levels (mg/l), (B) PMN counts (G/L), (C) platelet counts (G/L), and (D) hemoglobin level (g/l) before and nadir 1-3 weeks after anti-IL-6. CRP, C-reactive protein; IL-6, interleukin 6; PMN, polymorphonuclear leukocyte.

Biological signs of inflammation were present in all patients. CRP increase (100% of patients, median 212 mg/l), increased PMN counts [*n* = 26, 74% of patients, including 20 (57%) and 6 (17%) patients with leukemoid reactions and PMN counts over 10 and 20 G/L, respectively], thrombocytosis [*n* = 17 (48%)], and inflammatory anemia [*n* = 33 (94%)] (Table 1 and Figure 1). Improvement in the biological parameters was observed in all categories of patients: in previously untreated patients, in patients receiving concomitant systemic therapy, and in patients treated with tocilizumab only in the palliative setting (Supplementary Figure S1, available at https://doi.org/10.1016/j.esmoop.2024.104088). Increased IL-6 plasma levels were observed in all 13 patients in whom they were measured (median 141 pg/ml, range 58-680 pg/ml).

### Impact of tocilizumab on clinical and biological inflammatory syndrome

Tocilizumab was given once in 23 (66%) patients. In 12 patients receiving more than one course, treatment was administered on day (d) 0, d15, and d28 and then monthly in 8 patients, and monthly in 4 patients.

In six sarcoma patients with localized disease, tocilizumab was given to alleviate inflammatory syndrome causing fever and an alteration of PS contraindicating the surgical intervention needed to remove the primary tumor. Three, two, and one patient received one, two, and four courses before definitive surgery, respectively. All were operated and all are alive without progression at the time of the analysis. In advanced cancers, 20 of the 29 patients received only one course of tocilizumab, mostly because of rapid cancer progression.

All but one (97%) patient experienced resolution of fever and night sweats, with occasional body temperature inferior to 36°C. The last patient passed without temperature documentation. Weight was stable 3-4 weeks after tocilizumab in 21 (60%) patients, with 7 (20%) patients losing ≥1 kg. Seven (20%) patients gained weight (≥1 kg) 3-4 weeks after the first tocilizumab injection, including four of the nine (44%) patients who had lost >10% of weight (4/9 versus 3/26, *P* = 0.03).

All patients experienced a decrease in serum CRP levels after tocilizumab administration (median reduction −94%, range −8% to −99.5%, Wilcoxon *P* < 0.0001). Sixteen (44%) had normalized (<6 mg/l) CRP levels (Figure 1).

After tocilizumab, 30 (85%) patients had decreased PMN counts (median reduction −4.160 G/L, range −40.050 to +17.200 G/L, Wilcoxon *P* < 0.0001). Twelve of 26 (46%) patients with increased baseline PMN had normalized PMN counts (Figure 1).

After tocilizumab, 33 (94%) patients had decreased platelet counts (median reduction −192 G/L, range −591 to +177 G/L, Wilcoxon *P* < 0.0001); 16 of 17 (94%) patients with thrombocytosis had normalized platelet counts (Figure 1). The last patient has no documentation of platelet count after tocilizumab.

After tocilizumab, 27 (77%) patients had increased Hb levels (median Hb increase +17 g/l, range −30 to +48 g/l, Wilcoxon *P* < 0.0001) transiently (Figure 1). Two patients (6%) had increased Hb levels >120 g/l without red cell transfusion.

**Figure 2:**
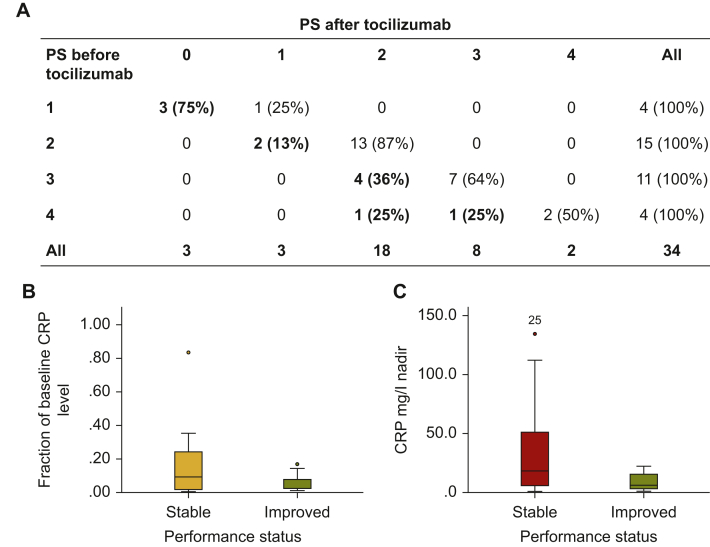
PS before and after tocilizumab administration. (A) Changes in PS after tocilizumab administration. (B) Magnitude of CRP level changes after tocilizumab administration in patients with stable and improved PS. (C) Nadir of CRP after tocilizumab administration in patients with stable and improved PS. CRP, C-reactive protein; PS, performance status.

An improvement in PS was observed in 11 (31%) patients (Figure 2A). Patients with improvement of at least 1 point of PS had more often a normalized serum CRP level after tocilizumab (8/11 versus 8/24, *P* = 0.03), and, overall, a greater magnitude of reduction of CRP levels (−94.3% ± 1.7% versus 84.7% ± 3.9%, *P* = 0.042, Figure 2B and C).

No specific side-effects related to tocilizumab administration^32^ were reported, in particular no skin, lung, or mycobacterial infection.

**Figure 3:**
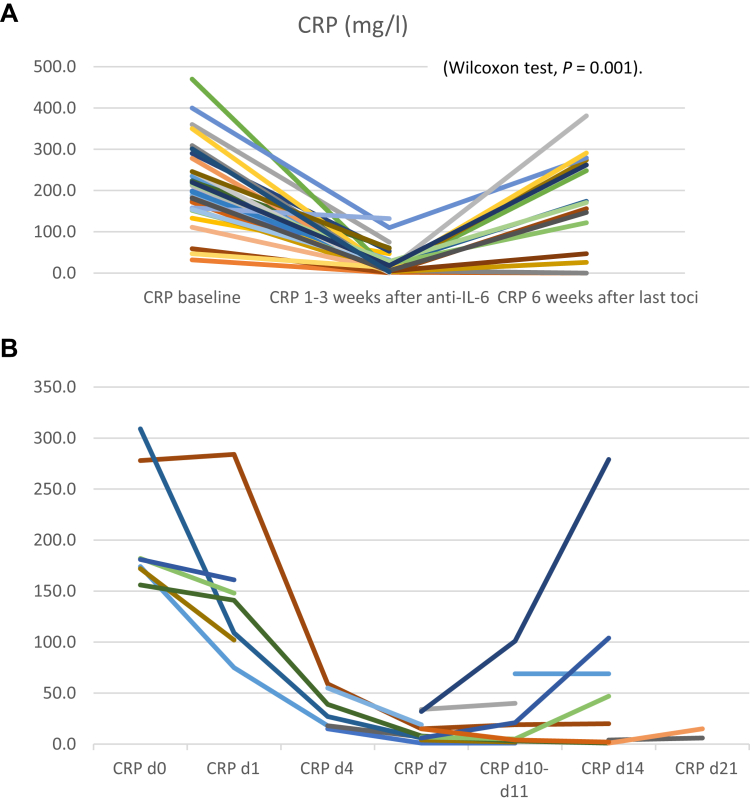
CRP levels before, during, and after tocilizumab. (A) In all patients: baseline, nadir, and 4-6 weeks after the last injection. (B) In patients with documented CRP levels within 21 days after the first tocilizumab injection. CRP, C-reactive protein; d, day.

The correction of inflammatory symptoms was transient. Four to six weeks after the last administration of tocilizumab, most patients in whom complete remission was not obtained had recurrence of clinical and biological symptoms in particular CRP increase, comparing CRP levels at 1-3 weeks after the first tocilizumab versus 6 weeks after the last tocilizumab injection (Figuren 3A, Wilcoxon *P* = 0.001). In patients followed sequentially, the nadir of CRP occurred at d7 after tocilizumab injection. CRP increased in some patients after this date, while others had a more prolonged CRP decrease (Figure 3B).

### Survival

All six sarcoma patients with localized disease were operated for the primary tumor, and all are alive disease free without PIS.

**Figure 4:**
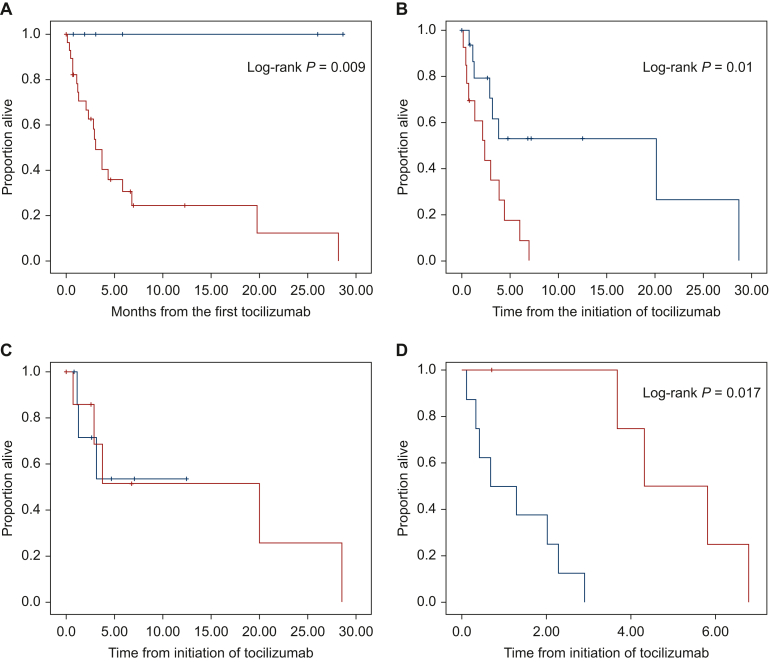
Survival from the date of tocilizumab treatment. (A) In advanced versus localized patients; (B) in patients with CRP levels above or under the median of the group; (C) in patients with CRP less than the median, with and without tocilizumab treatment; and (D) in patients with CRP higher than the median, with and without tocilizumab treatment.

The median survival of patients with PIS in advanced disease was 4 months (Figure 4A). For patients with advanced disease, CRP levels above the median of the group (212 mg/l) were associated with a worse survival (Figure 4B). In this latter subgroup, the normalization of CRP levels after tocilizumab was associated with a superior though short survival (median 4.3 months versus 0.6 months); this was not observed in the group with baseline CRP lower than the median of the group (Figure 4C and D).

No detrimental impact on disease progression or death was reported in this retrospective study. The six patients receiving tocilizumab with PD-1/PD-L1 Ab had a median survival of 19 months versus 3 months for those receiving tocilizumab with cytotoxic chemotherapy or best supportive care (BSC) only (*P* = not significant, not shown).

## Discussion

The objective of the study was to investigate the anti-inflammatory activity of the anti-IL-6R Ab tocilizumab in patients with solid tumors and PIS. The present series is the largest reported to our knowledge addressing the treatment of patients with PIS. It shows the significant therapeutic value of the blockade of IL-6 pathway for patients with PIS caused by solid cancers.

All 35 patients had a severe PIS. This population was frail with 100% presenting paraneoplastic fever, a median PS of 2, recent weight loss, and a major increase in CRP levels. Tocilizumab was an efficient symptomatic treatment for PIS in all 35 patients. Tocilizumab improved clinical symptoms, in particular fever in 97% of patients, but also improved PS in one-third of patients, enabled weight gain in patients with >10% body weight loss, and enabled the application of curative cancer treatments in patients previously contraindicated for curative surgery.

In solid tumors, PIS is rare but associated with a higher risk of progression and death in a large number of series.^1^, ^2^, ^3^, ^4^^,^^8^^,^^9^^,^^11^, ^12^, ^13^, ^14^ It is a severe and difficult-to-treat condition, whose resolution is obtained mostly when complete remission can be obtained. In this series, all 13 tested patients had increased serum IL-6 levels; CRP was also consistently elevated, with a median level before tocilizumab of 212 mg/l. CRP and IL-6 levels have been reported to be correlated in many series and different cancer types.^3^^,^^4^^,^^8^^,^^9^ CRP normalization is a biomarker to assess the activity of anti-IL-6 *in vivo*, confirming previous case reports and older studies with first generations of anti-IL-6 Ab.^6^^,^^22^^,^^24^^,^^27^^,^^28^

Tocilizumab is an anti-IL-6R Ab developed for the treatment of autoimmune diseases, in particular rheumatological diseases.^33^ It is also an active treatment for CRS associated with CAR-T-cell injections.^34^ It has been also used in case reports to treat PIS in patients.^27^^,^^28^ Thirty years ago, a mouse monoclonal anti-IL-6 Ab was used to treat hematological cancers and renal cell carcinoma with significant activity on inflammatory symptoms and occasional antitumor activity, but limited duration of activity with the development of anti-mouse Ab.^6^^,^^22^, ^23^, ^24^^,^^35^^,^^36^

Clinically, a resolution of paraneoplastic fever was observed in all 34 documented patients, with weight gain and improvement in PS observed in >30% of patients despite the short duration of treatment. The correction of biological hallmarks of inflammation was observed in all patients. CRP decreased massively in all patients and normalized in 46% of patients. Thrombocytosis was corrected in all 16 documented patients. The monitoring of CRP levels in some patients enabled to measure the activity of anti-IL-6R in this series: with a nadir of CRP levels 7-10 days after tocilizumab, some patients presented a slow increase beyond this date. Indeed, the duration of correction was limited in patients who received only one injection of tocilizumab. In this series, a single injection of tocilizumab was often proposed in the late advanced phase, in six patients receiving BSC only with poor life expectancy. Given the limited duration of CRP normalization after a single injection of tocilizumab, we now propose repeated 2-weekly injections of tocilizumab for patients with PIS who improved partially after a single injection to explore this question, with monitoring of CRP.

Some patients derived less benefit from tocilizumab injection, without PS improvement, and/or absence of normalization of CRP. This also could be related not only to a too short duration of treatment, but also to the possible role of other proinflammatory factors.^1^^,^^16^ When clinical symptoms of PIS, in particular fever, were not recurring, subsequent injections were often postponed. It can be recommended if benefit is observed to administer at d0, d14, and d28 and then monthly injections to obtain a more prolonged improvement in clinical and biological symptoms.

Sarcoma and lung cancer were the most common histological subtypes represented in this series.

In sarcoma, dedifferentiated liposarcoma was the predominant histotype in line with a recent preprint publication^37^, but other sarcoma histotypes (undifferentiated pleomorphic sarcoma, angiosarcoma) were also represented in this series. PIS is observed in a variety of solid cancers,^1^^,^^4^^,^^8^^,^^9^^,^^16^ including renal cell carcinoma, mesothelioma, and colorectal cancer among other cancer types. Anti-IL-6R treatment could be investigated in these tumors as well, noting that anti-IL-6 was successfully used for this purpose in advanced renal cell carcinoma with documented IL-6 production by tumor cells.^4^^,^^6^^,^^36^

There were three clinical situations where tocilizumab was used in this series.

Firstly, tocilizumab was used in patients with a localized operable disease, here only sarcomas. In those patients in whom PIS precluded surgical intervention, a transient tocilizumab treatment enabled a resolution of fever and improvement in clinical status allowing for surgical resection which enabled the complete resection of the primary tumor in all cases. PIS did not recur after complete surgical removal of the disease.

Secondly, tocilizumab was used in patients with advanced cancers receiving a systemic anticancer treatment, cytotoxic agents, or PD-1/PD-L1 Abs; when PIS deteriorated the clinical condition, a transient administration of tocilizumab enabled to alleviate clinical symptoms in all patients.

Thirdly, tocilizumab was used in patients in the advanced phase receiving only palliative care and no specific treatment. As expected, the life expectancy of the latter group was short.

The survival of the group of patients in the advanced-phase population was short, as expected for patients with a severe PIS. Importantly, in patients with the highest CRP level (above median), those in whom tocilizumab administration normalized CRP had a median survival; the survival was significantly superior to that of the other patients, though still short.

This study has several limitations. It is a retrospective collection of data, though exhaustive in the institution; therefore, the collection of potential adverse events may be imperfect. The duration of tocilizumab administration was variable. The population were heterogeneous in terms of tumor types and clinical situation. Patients were selected on the basis (among other criteria) of CRP increase; together with the limited number of patients tested for IL-6 levels, this limits our capacity to explore the correlation between CRP and IL-6 levels. We are now prospectively collecting these information for the patients candidate for tocilizumab treatment for a diagnosis of PIS. Nevertheless, this is the largest series of patients with PIS caused by a solid tumor treated with an anti-IL-6R Ab to our knowledge. The magnitude of clinical improvement observed in all patients and its capacity to enable curative treatment support the clinical and therapeutic utility of the treatment of PIS in patients with solid cancers.

In conclusion, tocilizumab is an efficient symptomatic treatment for PIS. It improved clinical symptoms, in particular normalized fever, enabled PS improvement in one-third of patients, and enabled curative surgery in patients previously contraindicated for intervention. It is also an interesting palliative treatment in end-stage patients to alleviate symptoms, including fever, fatigue, and cachexia. This approach deserves further exploration in patients with localized or advanced cancers with PIS.

## Funding

This work was supported and funded by academic grants, NetSARC+ (INCA and DGOS) and RREPS (INCA and DGOS), RESOS (INCA and DGOS), LYRICAN+ [grant number INCA-DGOS-INSERM 12563], InterSARC+ (INCA), LabEx DEvweCAN [grant number ANR-10-LABX-0061], EURACAN [grant number EC 739521], SHAPEMED, la Fondation ARC, Infosarcome, Ligue de L’Ain contre le Cancer, and La Ligue contre le Cancer (no grant number).

## Disclosure

The authors have declared no conflicts of interest.
