# Supplementary material for: Anti-IL-6R Ab tocilizumab to treat paraneoplastic inflammatory syndrome of solid cancers
Source: ESMO Open. 2025 Jan 3;10(1):104088. doi: 10.1016/j.esmoop.2024.104088 (PMC11758126; doi:10.1016/j.esmoop.2024.104088)
Supplement: Supplementary Figure 1 [file mmc1.docx]

**Supplementary Figure 1 : Evolution of PS and biological parameters after tocilizumab in three groups of patients**

**Localized disease (N=6)**  **Advanced disease receiving Advanced disease receiving**

**cancer systemic therapy (N=23)** **only best supportive care (N=6)**
